# Design and Evaluation of a Spoke-Based Double-Lumen Pediatric Gastrostomy Tube

**DOI:** 10.3390/children11020263

**Published:** 2024-02-19

**Authors:** Mihika Aedla, Charlotte J. Cheng, Anson Y. Zhou, Siya Zhang, Jocelyn Hsu, Katherine Hu, Jason C. Qian, Kevin Van de Sompel, Anthony Ho, Karun V. Sharma, Elizabeth A. Logsdon

**Affiliations:** 1 Department of Biomedical Engineering, Johns Hopkins University, Baltimore, MD 21218, USAazhou13@jhu.edu (A.Y.Z.); khu10@stanford.edu (K.H.); ksompel1@jhu.edu (K.V.d.S.); elogsdo1@jhu.edu (E.A.L.); 2 College of Medicine, Ohio State University, Columbus, OH 43210, USA; 3 School of Medicine, Stanford University, Stanford, CA 94305, USA; 4 School of Medicine, University of California San Diego, La Jolla, CA 92093, USA; 5 Department of Interventional Radiology, Children’s National Hospital, Washington, DC 20010, USA; kvsharma@childrensnational.org

**Keywords:** pediatric feeding, gastrostomy tubes, displacement, medical device, design innovation

## Abstract

Gastrostomy tubes (G-tubes) are the gold standard for feeding assistance for children with feeding dysfunction. Current G-tubes pose complications that interrupt the delivery of feed, including tube displacement and difficulty of at-home use. This study details an alternative, spoke-based, double-lumen G-tube design and preliminary validation of its function and usability. Pull force testing was performed on spoke G-tube models across three sizes and two classifications (hard/soft). Preliminary models were evaluated against market standards. Though the pull force of the spoke model was found to be lower than that of both market standards, hard modifications to the spoke model improved retentive force. Ease of use was tested amongst users unfamiliar with G-tube placement. The spoke design required 12.3 ± 4.7 s to deploy, less than half the time required for market standards. However, balloon G-tubes were still perceived to be easiest to use by 70% of participants, with indications that a spoke design may be easier to use if sized similarly to current G-tubes, with auxiliary improvements to factors such as grip. While there is a need for improvements in the material properties and manufacturing of the proposed design, this study provides early validation of the potential to address complications of existing G-tubes.

## 1. Introduction

G-tube feeding, the delivery of sustenance and nutrition via placement of a tube directly into the stomach through the abdominal wall, remains the gold standard for the assisted delivery of feed in patients with long-term feeding dysfunction. This population encompasses patients with an inability to feed independently through traditional oral access for longer than three months [1]. G-tube feeding is indicated across a broad range of conditions, ranging from cerebrovascular accident and congenital neurological disease to head-and-neck cancer [2].

Though G-tubes are effective in delivering feed when fully secure and functional, modern G-tubes are associated with a variety of complications that preclude reliable delivery. G-tube displacement is of notable concern, with reported rates as high as 7% even in largely sedentary or vegetative patients and rates estimated to be much higher in patients with hyperactivity or altered mental status [3]. In addition, displacement may be linked to other complications of varying severity, all of which can necessitate replacement procedures or emergent care [4].

Tube displacement is particularly dangerous in the pediatric feeding-assisted population. In addition to reported displacement rates of 22%, there are often accompanying complications, such as infection, which necessitate emergency admission [4,5]. Such barriers to reliable feeding in a population undergoing a period of critical growth and development engender the risk of serious long-term physical and neurological complications. It is, therefore, of considerable interest to make improvements in current G-tubes to mitigate tube displacement and associated complications.

G-tubes typically consist of an anchor to the stomach interior, a bolster to the stoma exterior, a tube for delivering food, and a feed port for inserting food [6]. Current gastrostomy tubes have various mechanisms for securing the tube inside the stomach, including balloons (i.e., MIC-Key Button G-Tube) and non-balloons (i.e., AMT Mini-One Non-Balloon Button G-Tube). The majority of G-tubes used in clinical practice today fall under the balloon button class, named for the water-inflatable balloon that retains the tubes to the gastric wall. Although these tubes are widely utilized because of their ease of use and ability to be replaced at home, their retentive balloons are prone to deflation, with degrees of severity ranging from partial water loss to complete bursting of the balloon [7]. Non-balloons are the typical alternative and rely on a highly stable internal retention mechanism that does not require inflation; however, these tubes are infrequently placed because of the complexity of their placement mechanism, which involves an external stylus tool, high amount of force required to place, irritation at the stoma site, and an inability to be replaced at home [8]. These shortcomings may lead to hospitalization, ineffective feeding, infection, and general discomfort [9].

In this paper, we discuss alternative G-tube designs that aim to address internal retention instability and poor ease of use via a spoke-based, double-lumen internal retention mechanism that cannot deflate, does not require additional apparatuses, and reduces excessive handling force. We provide preliminary validation of the design and function of a spoke geometry, highlighting the proposed design’s function and value, the potential to address prevailing complications with current gastrostomy feeding, and key future areas of modification for improvement.

### 1.1. Device and Testing Rig

#### 1.1.1. G-Tube Design

**Figure 1:**
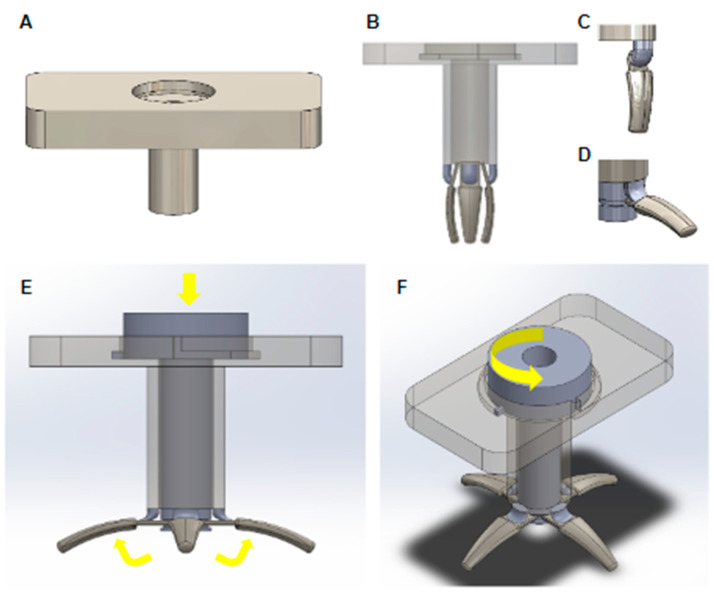
Spoke G-tube design. (**A**) Outer tube without spokes. (**B**) Inner tube inserted into the outer tube with spokes forming a double lumen. (**C**) Spokes in retracted conformation for insertion of removal. (**D**) Spokes in expanded conformation for retention. (**E**) Spokes shift from retracted to expanded form by inserting the inner tube into the outer tube; the base of the inner tube pushes against tabs at the base of the spokes, causing them to splay out. (**F**) The inner tube twists and locks in place via a perpendicular tab that follows a track inside the flange of the outer tube.

The proposed G-tube encompasses a simple and novel design to enhance ease of insertion and removal for pediatric patients, with models sized at 14 Fr. The tube segment consists of concentric, double-layered cylinders, forming a double-lumen structure when assembled (Figure 1). G-tubes are typically inserted percutaneously via endoscopy, in which an endoscope is inserted from the mouth to the stomach, the stomach is inflated with air, and a small incision is made on the left side of the abdomen and stomach. Our proposed design allows the outer tube, fitted with a flange, to be inserted into the stomach directly through an abdominal incision until the flange sits comfortably on the outer abdomen this aspect of the insertion is consistent with placement procedures for existing G-tubes. The inner tube may then be positioned through the outer tube, deploying an expansion-retraction mechanism (described below) inside the stomach to fix the outer tube to the gastric wall.

#### 1.1.2. Expansion-Retraction Mechanism

The distal end of the outer tube consists of four equidistant, rigid, appendage-like spokes connected to the tube wall by flexible strips made of flexible polymer. At the wider base of each spoke is a small rectangular tab fitted within the lumen of the outer tube at an acute angle. In the retracted conformation (without the inner tube inserted inside the outer tube), the spokes point downwards in a relaxed state. During insertion of the inner tube, the bottom of the inner tube will press on the tabs of the rigid spokes, causing the spokes to deploy and splay out perpendicularly to the bottom of the tube. In this expanded state, the G-tube is retained against the gastric wall (Figure 1).

#### 1.1.3. Testing Rig: Biofidelic Stomach Model

Biofidelic stomach models were developed with a combination of Ecoflex and Dragonskin to assess prototype function. A mixture of 0.5 cm thick Ecoflex 0030 representing the abdominal wall and 0.5 cm Dragonskin representing the stomach was prepared for all testing models.

## 2. Materials and Methods

### 2.1. G-Tube Manufacturing

The prototypes were modeled in SolidWorks and 3D printed using various methods and materials. Tubes of varying sizes (14, 24, and 36 Fr) were tested to evaluate the spoke-based internal retention mechanism’s contribution to the retentive ability of the tube without the manufacturing limitations of the small 14 Fr tubes. The 14 Fr and 24 Fr tubes were tested against competitor tubes, while the 36 Fr tube was used as an initial, larger-scale proof-of-concept (POC) even as no competitor comparisons exist at this size.

Iteration #1—Large-scale POC prototypes: The initial 36 Fr scale POC prototypes were printed in thermoplastic polyurethane (TPU) using selective laser sintering (SLS) through the 3D printing company Shapeways.

Iteration #2—Scale static-form prototypes: In order to finely adjust the flexibility of the prototype, to-scale 14 Fr prototypes were manufactured by Johns Hopkins Carnegie Center for Surgical Innovation, using the material jetting capabilities of the Stratasys Objet500 Connex3 Multi-Material 3D printer. This iteration involved a combination of Vero Clear, a rigid material, and Agilus 30, a rubber-like material, in different ratios across the components of the device: 3:1 Vero/Agilus for the body, 3:1 Vero/Agilus for the spokes, and 1:3 Vero/Agilus for the flexible strips connecting the spokes to the outer tube. The adjustment of flexibility allowed the prototype to improve its flexibility while retaining the strength required for increased function. This iteration was purely an assessment of material modulation and was ultimately not tested because of manufacturing limitations that prevented full expansion and retraction.

Iteration #3—Dynamic-form prototypes: To better simulate scale prototypes made of medical-grade silicone that are able to expand/retract, this iteration was manufactured via silicone 3D printing. The prototypes were printed with Spectroplast patented methodology Silicone Additive Manufacturing (SAM) in their proprietary TrueSil material, with iterations ranging from Shore 20A to Shore 60A.

Iteration #4—Dynamic-form prototypes with hardness modification: To better simulate the scale prototype in a hard material, a 14 Fr spoke G-tube was created. The outer and inner lumens were printed on a Stratasys F370 3D printer. The spokes were created using wire electrical discharge machining and assembled to the outer lumen using aluminum wire. The 24 Fr hard modification utilized the TrueSil Shore 20A prototype with strips of aluminum attached along the spokes for greater stiffness. The 36 Fr hard modification utilized the same manufacturing methods as described in Iteration #1, given that TPU is a comparably hard material for the purposes of this stage of testing.

### 2.2. Biofidelic Stomach Model: Development and Validation

**Figure 2:**
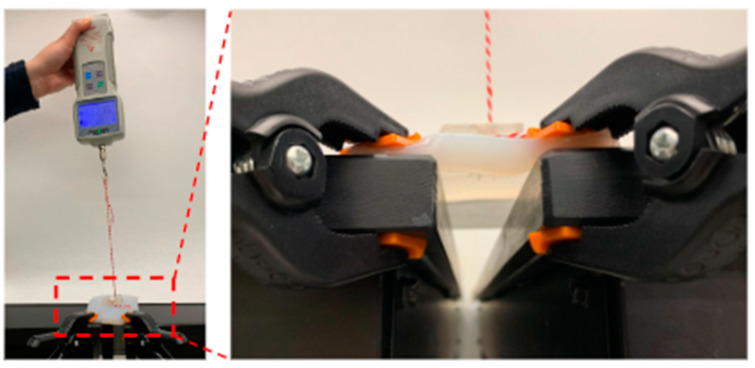
Biofidelic testing rig.

To determine the most biofidelic model for functional tests, a series of stomach models were created and compared with a 1 cm porcine belly slab, as previous studies have shown similar mechanical properties between porcine skin and digestive tissue [10,11]. All stomach models were created with Ecoflex 0030 and Dragonskin (Figure 2), as their efficacy for biofidelic human tissue modeling has been well documented in the literature, in which silicone has been shown to have similar properties to human organ tissue [12,13,14]. Expanded state pull tests were conducted, and the results were compared with the porcine belly slab to determine the most biofidelic model. Using a non-balloon for a pull test, the stomach model with the most similar pull force to the porcine model (15.5 N) was selected for the pull test study. The combination most similar to the porcine belly slab, determined to be a 1 cm thick, 1:1 ratio (14.8 Fr) of Ecoflex and Dragonskin, was selected for prototype testing.

### 2.3. Expanded State Pull Test

Pull tests were conducted to assess the force required to completely displace the deployed G-tube out of a neonatal stomach model. An 8 cm × 8 cm × 1 cm stomach model (Figure 2) was made using 1:1 Ecoflex 0030 and Dragonskin 50% Dragonskin and 50% Ecoflex. The opposite sides of the stomach model were clamped to the sides of two tables with a 2-inch gap in between (Figure 2). Using a scalpel, a 1 cm incision was made to the center of the stomach model. A 6 Fr dilator was inserted and removed. Dilators at 2 Fr intervals were inserted and removed until the size of the G-tube was reached at 14 Fr. The G-tube was then inserted in the retracted state, followed by the internal retention mechanism deployment into the expanded state. One end of a 20 cm string was attached under the outer flange of the G-tube. An overhand loop knot was tied on the other end of the string with 10 cm of string between the two knots. The loop knot was hung around the hook attachment of a Sisco 0–500 N digital force gauge. The force transducer was pulled upwards at a rate of approximately 1 cm/s. The trial was stopped when the G-tube was completely removed from the stomach model, and the highest recorded force was noted.

### 2.4. Retracted State Pull Test

A retracted state pull test was conducted to assess the force required to fully remove the feeding tubes from a neonatal stomach when the retention mechanism is not deployed. The same pull test protocol was used for the retracted state pull test as in that of the expanded state.

### 2.5. Usability Test

**Figure 3:**
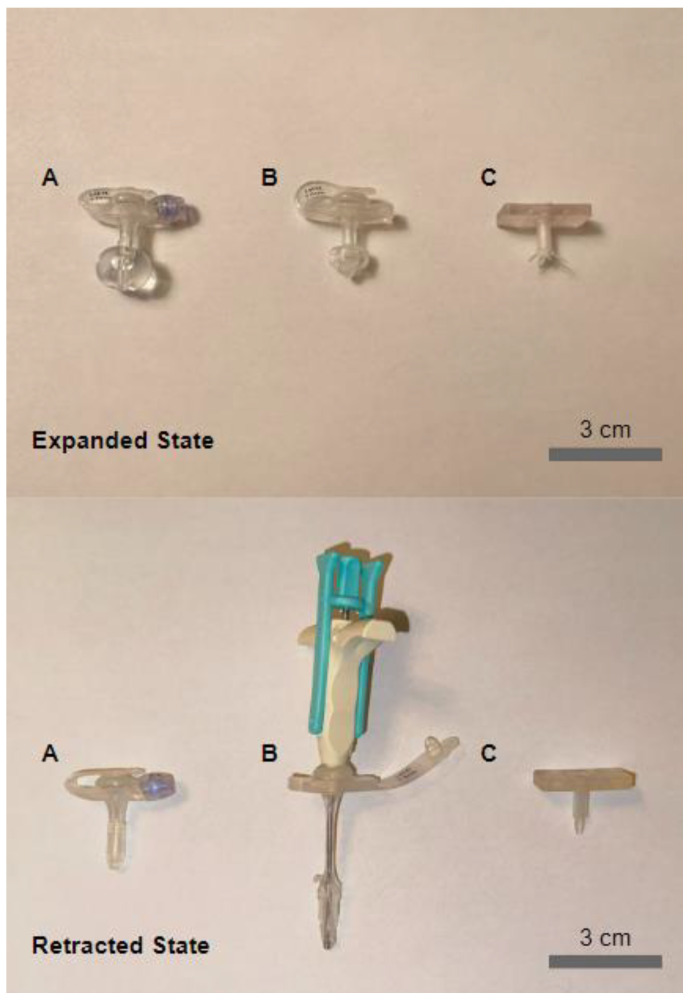
G-tubes tested. (**A**) Balloon, (**B**) Non-balloon, and (**C**) Spoke-based G-tubes in their expanded and retracted conformations.

A usability test was conducted to assess each G-tube’s (Figure 3) ease of use and potential adoption based on user performance and perception data. The participants were shown three G-tubes (balloon, non-balloon, and spoke) encoded as A, B, and C and were asked to answer a question regarding their initial impression of the ease of use. The participants then watched brief instructional videos (Supplementary Videos S1–S3) explaining the procedure for each G-tube deployment (expansion) and retraction. For the balloon and non-balloon, the instructions were modeled after instructions provided by manufacturers in their respective manuals. The participants were then asked to expand and retract a randomly selected G-tube while being timed. After completing the first attempt, the participants were asked to complete a second attempt with the same G-tube. The set of two tests was conducted in random order for all three different G-tube models. After completing all six tests, participants were asked to complete a brief survey (Supplementary Document S1) to assess their perceptions following performance testing.

## 3. Results

### 3.1. Expanded State Pull Test

**Figure 4:**
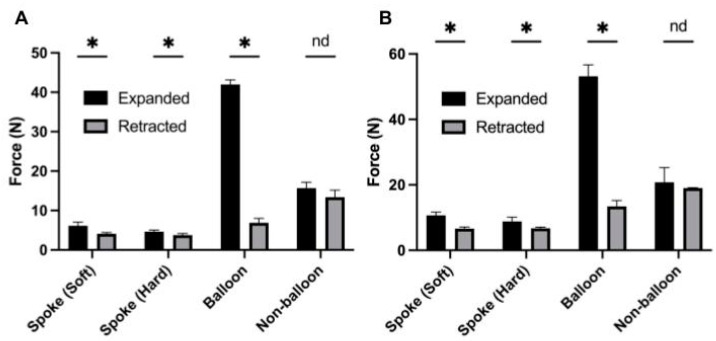
Pull and Resistance force test results for 14 Fr and 24 Fr G-tubes with varying designs and hardnesses (*n* = 5). The highest force withstood by each G-tube in an open (Expanded) and closed (Retracted) confirmation was recorded. Hard spoke, soft spoke, balloon, and non-balloon designs were tested in (**A**) 14 Fr and (**B**) 24 Fr sizes. The soft spoke was manufactured with silicone, while the hard spoke design was reinforced with hard metals or plastics. * *p* < 0.05 indicates significance measured by *t*-test. nd = no statistically significant difference.

Pull tests were conducted to evaluate the amount of force required to displace a G-tube from the stomach model when deployed in the expanded state. For the spoke design, the 14 Fr G-tube made with soft material withstood 4.66 N of force, while the 24 Fr withstood 8.84 N of force (Table 1, Figure 4). The spoke G-tube manufactured with hard material withstood 6.16 N for the 14 Fr tube, 10.66 N for the 24 Fr tube, and 20.14 N for the 36 Fr tube. Competitor tubes were also tested using the same protocol. The 14 Fr balloon design withstood 41.97 N, while the 24 Fr version withstood 53.17 N. The non-balloon design withstood 15.70 N for the 14 F size and 20.77 N for the 24 F size (Table 1, Figure 4). Soft spoke 36 Fr results were not included as they could not be inserted into the stomach model. The soft material was insufficiently rigid to hold its shape while being inserted.

### 3.2. Retracted State Pull Test

Pull tests were used to assess the amount of force required to remove the G-tube from the stomach model while the tube was in its retracted state. Our spoke model made with soft material required 3.76 N to displace the 14 Fr tube and 6.70 N to displace the 24 Fr tube (Table 1, Figure 4). The spoke design manufactured with hard material required 4.12 N to displace the 14 Fr tube and 6.62 N to displace the 24 Fr tube. The balloon G-tube required 6.9 N to displace the 14 Fr tube, while the 24 Fr tube required 14.03 N. The non-balloon tube required 13.40 N to displace the 14 Fr tube and 19.03 N to displace the 24 Fr tube (Table 1, Figure 4). Soft spoke 36 Fr results were not included as they were unable to be inserted in the stomach model. The force difference between the expanded and retracted state pull tests was calculated. The results for the soft material spoke, hard material spoke, and the balloon at the 14 Fr size was found to be significant (*p* = 0.001449, *p* = 0.006108, and *p* = 0.000006, respectively). Similar comparisons at the 24 Fr size were also found to be significant (*p* = 0.000064, *p* = 0.005039, and *p* = 0.000064, respectively). Non-balloon differences in force were found to be not significant at both sizes.

### 3.3. Usability Test

For each of the G-tubes (balloon, non-balloon, spoke), random college-aged participants with no experience in G-tube use were asked to perform an expansion and retraction procedure, twice. Across the performance results in the second trial, the order of fastest completion was spoke, balloon, and non-balloon at 12.3 ± 4.7, 28.9 ± 8.8, and 30.0 ± 9.2 s, respectively. The non-balloon had 2 participants fail to complete the procedure (take greater than 90 s without completing an expansion and retraction) in Trial 1 and three participants in Trial 2. Neither balloon nor spoke had any participants fail to complete the procedure for any trial (Table 2). In comparing the change in completion time between Trials 1 and 2, spoke had the greatest decrease at 49.0%, followed by balloon at 38.9%, and non-balloon at 33.4% (Table 2). The changes in completion time were found to be statistically significant in the spoke, balloon, and non-balloon (*p* = 0.006146, *p* = 0.001625, and *p* = 0.001625, respectively). 

Before and following the G-tube testing, participants were surveyed to understand their perceptions of using each device. The balloon had the greatest increase in perception of ease of use, increasing from being ranked as the easiest G-tube by 40% of participants pre-test to 70% post-test. Both non-balloon and spoke experienced decreases, dropping from 10% to 0% and 50% to 30%, respectively (Table 3). Across survey questions measuring median difficulty and median confidence in use, a similar trend was observed, with the balloon leading in being the least difficult and giving participants the most confidence to use, followed closely by spoke, with a steep drop to non-balloon (Table 3).

## 4. Discussion

### 4.1. Manufacturing

The results in this paper preliminarily indicate the capacity for a spoke retention mechanism to function, defined here as the ability to fully expand into the retentive position and fully retract for insertion and removal. With this, the spoke G-tube achieves the primary focus of this stage of iteration: demonstration of a novel and potentially easier-to-use mechanism. However, with this focus, the prototypes tested were constrained by limitations in funding and manufacturing capabilities. Prototypes were produced largely through 3D printing rather than more robust methods such as injection molding. Prototype manufacturing, therefore, faced challenges in material constraints. For example, prototypes needed to be produced from a limited range of plastics and silicone-like materials, and spokes could not easily be produced with multiple materials or encasings. These challenges resulted in complications with brittleness and multi-round testing, particularly at smaller scales. However, these are addressable through improvements in manufacturing capability and technical refinement. It is, therefore, the authors’ belief that with future improvement, the proposed spoke design may achieve efficacy comparable to competitor tubes while addressing the design flaws that give rise to common complications.

### 4.2. Pull Force

Pull force is a critical metric for a G-tube model’s capacity for retention and maximization of retentive capacity, which reduces the likelihood and frequency of unintended displacement. Pull force was studied on the tubes in both the expanded and retracted positions, and the difference in force was calculated to evaluate the magnitude of additional resistance conferred by the spokes compared with the natural retention of the tube material itself. Both the pull force and the delta force were found to increase with increasing G-tube size, suggesting that the contribution of the spokes to improve retention improved with larger, thicker spoke dimensions. Increasing the hardness of the spoke material was also found to improve retention, highlighting the potential of these designs to demonstrate improved retentive ability with improvements in material selection. Competitor balloon and non-balloon G-tubes were also tested at the 14 Fr and 24 Fr sizes for comparison. It should be noted that while there was a lack of significant change between the expanded and retracted states for the non-balloon button, this is attributable to the tube’s significant retentive ability, even when retracted, rather than an indication of its ineffectiveness upon deployment. The non-balloon’s retracted form is bulkier and sturdier than either the balloon button or the spoke G-tube; unlike the other designs, deployment of the non-balloon only inflates the tube minimally past its “retracted” form and is used largely to lock the tube in place. This contributes to its difficulty of use at home; parents and caretakers do not prefer the non-balloon to the balloon button, despite its strength [4]. Overall, although the spoke design in its current iterations did not achieve the pull force metrics of the competitor tubes at comparable sizes, the performance of the harder, reinforced spokes does suggest some potential for reaching the market standard with materials and manufacturing improvements.

The manufacturing methods used to produce these prototypes presented several challenges. Because the G-tubes were 3D printed, there was a limited range of materials in which they could be reproduced. Furthermore, the hardness of the material was unable to be significantly adjusted with these manufacturing methods. Thus, although our G-tubes were of similar stoma size to competitor tubes, they did not have the same stiffness and mechanical integrity. In future iterations, using the same fundamental design, we will work on reinforcing the retention strength of the spokes of the G-tube. In particular, we plan to modify the material base of the device with a higher modulus polymer and implant the core of the spokes with a stiff but flexible nitinol wire. Additionally, we intend to use alternative manufacturing methods, such as injection molding, in order to appropriately integrate materials with multiple properties together and modulate the material hardness to emulate current G-tubes on the market.

### 4.3. Usability

In commercially available balloon models, at-home insertion/removal can be difficult for caretakers. In non-balloon models, at-home replacement is typically not possible and requires endoscopic guidance under anesthesia or with topical pain reliever. Both models require an external device for insertion/removal, with a syringe and a specialized stylus being used for the balloon and non-balloon, respectively. It should be noted that while the balloon model is technically able to be replaced at home, caretakers still have a degree of discomfort with the process and may opt for the highly inconvenient alternative of having it replaced at the hospital [4]. We postulate that the necessity for an external syringe, which may not always be accessible, and the inflation of an occluded balloon in the stoma site contribute to this discomfort. Thus, in the spoke design, we eliminate the need for an external syringe/stylus. The entire insertion/removal process, including locking in place, can be completed with mechanisms intrinsic to the structure of the G-tube, with no need for inflation or stretching. We imagine that this mechanism will make at-home replacements by caretakers a simpler and less stressful process, thereby decreasing the frequency and need for hospital visits in case of displacement.

In conducting our usability study, while we found the spoke G-tube to have the shortest time to expand and retract as well as the greatest improvement in time decrease between trials 1 and 2, this evidence of a quicker procedure did not translate into participant perceptions of greater ease of use. Rather, the balloon G-tube, despite taking over two-fold the amount of time required for expansion and retraction, was considered the easiest to use by a majority of users following testing. Further, the balloon actually experienced an increase in the percentage of participants rating it as the easiest to use from pre-test (purely visual assessment) to post-test, from 40% to 70%. The spoke experienced a decrease from 50% to 30%.

Due to the material choice and size of the components for this iteration of the spoke prototype, we observed the difficulty of gripping amongst participants as a common issue. This qualitative feedback, coupled with the performance and perceptions data, drives us to believe that this decrease in the perception of ease of use for the spoke may be a result of the size and accessories of the prototype rather than discontent with the fundamental mechanism. Future iterations for this device will prioritize such factors to enhance usability.

While the usability study demonstrated the promise of our device in easing at-home replacement, there are several limitations to the study design. For one, the subjects of the study consisted of a random sampling of undergraduate students who have not had prior experience with pediatric G-tubes. In a future usability study, upon further refinement of our device, we will design the study to focus on at-home caretakers with prior experience with pediatric G-tubes in order to obtain data that are more relevant to our target user population. Future iterations of this study will also include a larger sample size to ensure robust and generalizable results. Nevertheless, we believe the study conducted provides convincing data to support the ease of use of the device.

Beyond user perceptions of ease of use, a new G-tube design will undoubtedly face clinical adoption challenges, particularly in a space such as pediatric gastrostomy feeding, which has not experienced significant innovation in decades. A key challenge will be regulatory approval. To that end, our design has been restricted to size and materials comparable to existing G-tubes in order to best align with the 510(k) approval pathway. Another key challenge will be instilling confidence in clinicians that the design will be adequate, such that they recommend them for use by caretakers in the home. Future usability studies with a more finalized prototype will also include clinicians to obtain their perspectives on the practicality of adoption.

## 5. Conclusions

This study provides initial validation of the potential of a spoke, double-lumen G-tube retention mechanism to address displacement and ease of use issues. While the results in this paper should be considered preliminary, the authors believe there is compelling evidence of the design’s ability to perform to market standard with good ease of use and minimized displacement risk upon improvement of the tube’s manufacturing methods, dimensions, and material rigidity.

## Figures and Tables

**Table 1:** Pull tests of the G-tubes in the expanded and retracted conformations were performed for various designs and manufacturing materials (*n* = 5). The highest force withstood during the pull test for the model with spokes in the expanded form (Pull-Expanded) and retracted form (Pull-Retracted) was recorded. The difference between the two forces was calculated (Delta). Hard spoke, soft spoke, balloon, and non-balloon designs were tested in 14 Fr and 24 Fr sizes. Hard spoke 36 Fr tubes were also tested, but the soft spoke 36 Fr results are not included (noted N/A) as the tubes were unable to be inserted in the stomach model. The soft spoke was manufactured with silicone, while the hard spoke design was reinforced with hard metals or plastics. Balloon and non-balloon tubes were manufactured with standard medical-grade silicone.

Tube	Size	Classification	Method of Manufacture	Material	Pull Test—Expanded (N)	Pull Test—Retracted (N)	Delta
Spoke	14 F	Soft	Silicone Additive Manufacturing	Medical Grade Silicone	4.66	3.76	0.90
Spoke	24 F	Soft	Silicone Additive Manufacturing	Medical Grade Silicone	8.84	6.70	2.14
Spoke	36 F	Soft	Silicone Additive Manufacturing	Medical Grade Silicone	N/A	N/A	N/A
Spoke	14 F	Hard Modification	Electrical Discharge Machining and Assembly	Acrylonitrile Butadiene Styrene and Aluminum	6.16	4.12	2.04
Spoke	24 F	Hard Modification	Silicone Additive Manufacturing and Aluminum Attachment	Silicone and Aluminum	10.66	6.62	4.04
Spoke	36 F	Hard Modification	Selective Laser Sintering	Thermoplastic Polyurethane	20.14	7.74	12.40
Balloon	14 F	Standard	Extrusion, Dip Coating, and Bonding	Medical Grade Silicone	41.97	6.90	35.07
Balloon	24 F	Standard	Extrusion, Dip Coating, and Bonding	Medical Grade Silicone	53.17	14.03	39.13
Non- balloon	14 F	Standard	Extrusion, Dip Coating, and Bonding	Medical Grade Silicone	15.70	13.40	2.30
Non- balloon	24 F	Standard	Extrusion, Dip Coating, and Bonding	Medical Grade Silicone	20.77	19.03	1.73

**Table 2:** Usability test performance data (*n* = 10). * *p* < 0.05 indicates significance measured by *t*-test.

G-Tube	Trial	Average Time (s)	Number Trials Incomplete	Time Difference between Trials (s)
[A] Balloon	Trial 1 Total	47.2 ± 14.2	0	18.3 *
	Trial 2 Total	28.9 ± 8.8	0	
[B] Non-balloon	Trial 1 Total	45.1 ± 6.9	2	15.1 *
	Trial 2 Total	30.0 ± 9.2	3	
[C] Spoke	Trial 1 Total	24.2 ± 13.7	0	11.9 *
	Trial 2 Total	12.3 ± 4.7	0	

**Table 3:** Usability test participant perception data (*n* = 10).

Question	Balloon	Non-Balloon	Spoke
Easiest to use (pre-test)	40%	10%	50%
Easiest to use (post-test)	70%	0%	30%
Top choice for at-home use (post-test)	70%	0%	30%
Median difficulty (1–10)	2.5	6	3
Median confidence with at-home placement (1–10)	6.5	3.5	6

## Data Availability

The data presented in this study are available on request from the corresponding author. The data are not publicly available due to to specific ethical and privacy considerations.

## Supplementary Materials

The following supporting information can be downloaded at: https://www.mdpi.com/article/10.3390/children11020263/s1, Document S1: Pediatric G-Tube: Post-Study Survey. Figure S1: Views of double-lumen G-tube design. (A) Surface wire frame view (B) Cross-sectional view (C) 3-dimensional view (D) Transparent 3-dimensional view. Videos S1–S3: MOV.
