# Supplementary material for: Design and Evaluation of a Spoke-Based Double-Lumen Pediatric Gastrostomy Tube
Source: Children (Basel). 2024 Feb 19;11(2):263. doi: 10.3390/children11020263 (PMC10888183; doi:10.3390/children11020263)
Supplement: Supplementary file 1 [file children-11-00263-s001.zip › Document S1.pdf]

Pediatric G-Tube: Post-Study Survey

1. Name

2. Date of Birth

Example: January 7, 2019

3. Major

4. Gender

5. Assuming equal durability and feeding function, which G-tube do you believe is most easy to use at first glance? [PRIOR TO TESTING]

Mark only one oval.

- G-tube A
- G-tube B
- G-tube C

6. Assuming equal durability and feeding function, which G-tube do you believe is most easy to use at first use? [AFTER TESTING]

Mark only one oval.

- G-tube A
- G-tube B
- G-tube C

7. How difficult was it to expand and retract G-tube A?

Mark only one oval.

	1	2	3	4	5	6	7	8	9	10	
Not											Extremely difficult

8. With some training (i.e, 5 times in a model), how confident would you be in placing G-tube A in a child in a home setting?

Mark only one oval.

	1	2	3	4	5	6	7	8	9	10	
Not	◯	◯	◯	◯	◯	◯	◯	◯	◯	◯	Extremely confident

9. How difficult was it to expand and retract G-tube B?

Mark only one oval.

	1	2	3	4	5	6	7	8	9	10	
Not	◯	◯	◯	◯	◯	◯	◯	◯	◯	◯	Extremely difficult

10. With some training (i.e, 5 times in a model), how confident would you be in placing G-tube B in a child in a home setting?

Mark only one oval.

	1	2	3	4	5	6	7	8	9	10	
Not	◯	◯	◯	◯	◯	◯	◯	◯	◯	◯	Extremely confident

11. How difficult was it to expand and retract G-tube C

Mark only one oval.

	1	2	3	4	5	6	7	8	9	10	
Not	◯	◯	◯	◯	◯	◯	◯	◯	◯	◯	Extremely difficult

12. With some training (i.e, 5 times in a model), how confident would you be in placing G-tube C in a child in a home setting?

Mark only one oval.

	1	2	3	4	5	6	7	8	9	10	
Not	◯	◯	◯	◯	◯	◯	◯	◯	◯	◯	Extremely confident

13. Which G-tube do you believe would be most easy to use after ~5 tries of practice? (RANK: 1 being most willing; 4 being least willing)

Check all that apply.

	1	2	3
**G-tube A**	☐	☐	☐
**G-tube B**	☐	☐	☐
**G-tube C**	☐	☐	☐

14\.

Given equal durability and feeding functionality across all G-tubes, which G-Tube would you be most willing to adopt for at home use in a child. (RANK: 1 being most willing; 4 being least willing)

Check all that apply.

	1	2	3
**G-tube A**	☐	☐	☐
**G-tube B**	☐	☐	☐
**G-tube C**	☐	☐	☐
