# Supplementary material for: Design and Evaluation of a Spoke-Based Double-Lumen Pediatric Gastrostomy Tube
Source: Children (Basel). 2024 Feb 19;11(2):263. doi: 10.3390/children11020263 (PMC10888183; doi:10.3390/children11020263)
Supplement: Supplementary file 1 [file children-11-00263-s001.zip › New Supplementary Addition.pptx]

## Slide 1
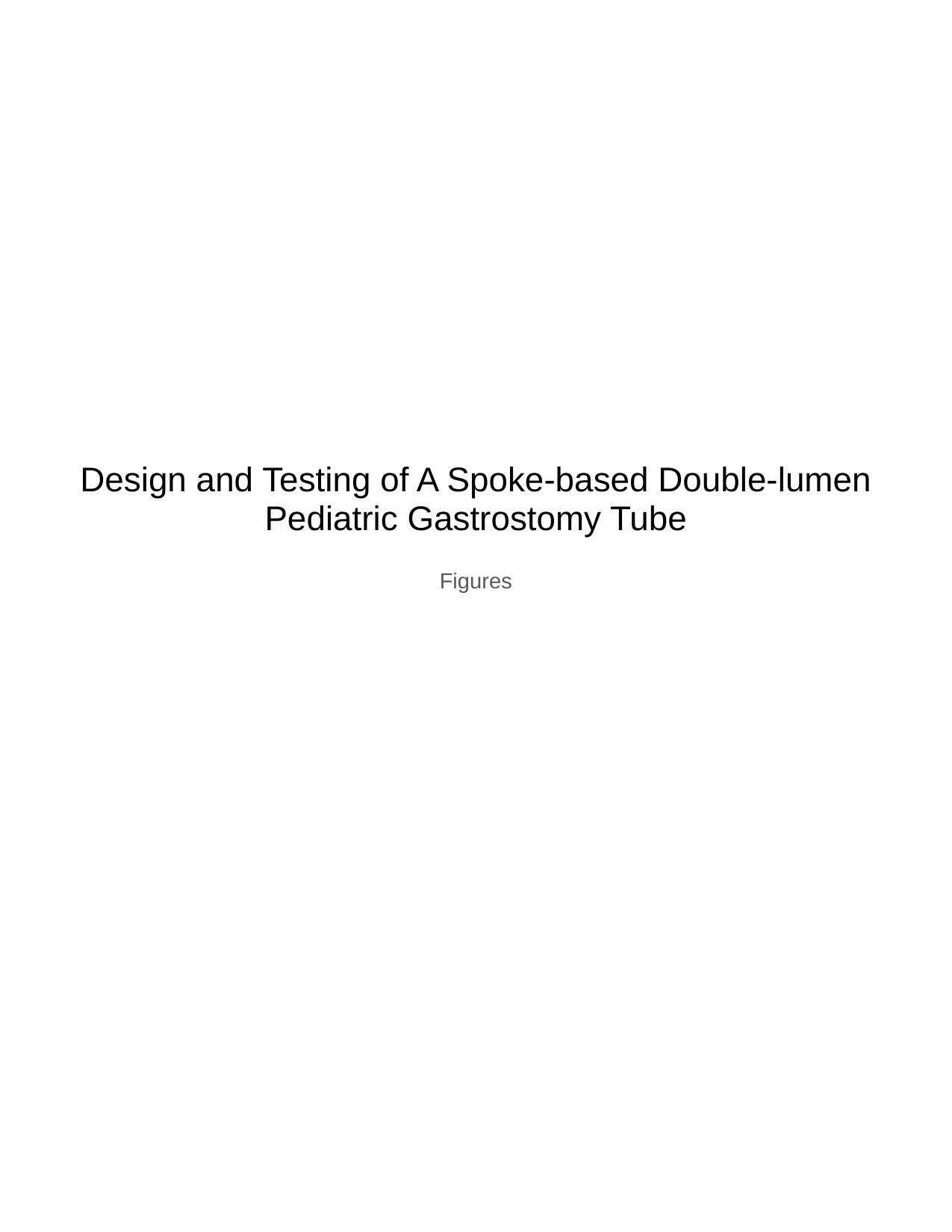

# Design and Testing of A Spoke-based Double-lumen Pediatric Gastrostomy Tube
Figures

## Slide 2
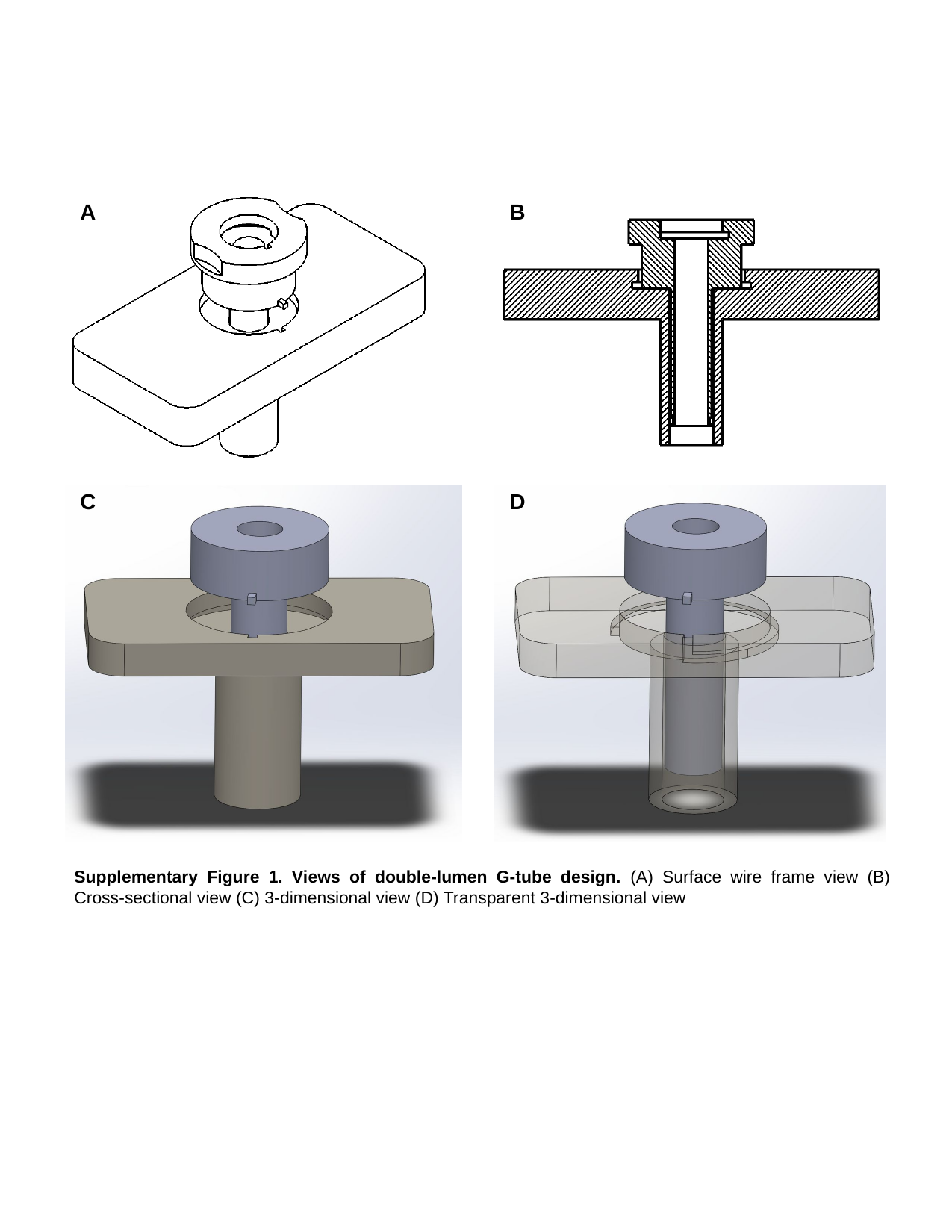

A
B
C
D
Supplementary Figure 1. Views of double-lumen G-tube design. (A) Surface wire frame view (B) Cross-sectional view (C) 3-dimensional view (D) Transparent 3-dimensional view
